# Association between dietary zinc intake and herpes simplex virus seropositivity in U.S. adults: a cross-sectional study

**DOI:** 10.1186/s12879-026-12927-1

**Published:** 2026-02-26

**Authors:** Chunhua Liu, Yingguo Liu, Jingjing Liu, Zhaoyong Lv, Yanan Qin, Mengpeng Li

**Affiliations:** 1 Department of Infection, Liaocheng People’s Hospital, Liaocheng, Shandong China; 2 Liaocheng Infectious Diseases Hospital, Liaocheng, Shandong China; 3 Pediatric Laboratory of Liaocheng People’s Hospital, Liaocheng, Shandong China; 4 Shandong Provincial Key Medical and Health Laboratory of Stem Cell and Regenerative Medicine Translation (Liaocheng People’s Hospital), Liaocheng, Shandong China

**Keywords:** Zinc, Herpes simplex virus, National Health and Nutrition Examination Survey, Epidemiology

## Abstract

**Background:**

Although recent research has demonstrated an association between serum zinc deficiency and susceptibility to various viral infections, the relationship between dietary zinc intake and herpes simplex virus (HSV) seropositivity remains unclear.

**Methods:**

The National Health and Nutrition Examination Survey (NHANES) (2007–2016) provides data on HSV-1 and HSV-2 status and dietary zinc intake. The associations between dietary zinc and HSV-1 and HSV-2 were evaluated via various statistical methods, including multivariate logistic regression, restricted cubic spline analysis, and subgroup analysis.

**Results:**

In total, 6,483 individuals were enrolled, with 58.9% (3,817/6,483) testing positive for HSV-1 and 19.3% (1,253/6,483) testing positive for HSV-2. After adjusting for all covariates in the multivariate logistic regression, compared with the lowest zinc intake group (Q1: <7.51 mg/day), the adjusted odds ratios (ORs) for HSV-1 and HSV-2 in the higher-zinc intake groups were as follows: for HSV-1, Q2 (7.51–10.87 mg/day) had an OR of 0.81 (95% confidence interval [CI]: 0.72–0.98, *p* = 0.027), and Q3 (10.87–15.63 mg/day) had an OR of 0.85 (95% CI: 0.70–1.03, *p* = 0.093), and Q4 (> 15.63 mg/day) had an OR of 0.84 (95% CI: 0.67–1.04, *p* = 0.114); for HSV-2, Q2 had an OR of 0.80 (95% CI: 0.64–1.00, *p* = 0.050), Q3 had an OR of 0.81 (95% CI: 0.64–1.02, *p* = 0.069), and Q4 had an OR of 0.75 (95.

**Conclusion:**

Dietary zinc intake exhibited a U-shaped association with HSV-1 and HSV-2 seropositivity, indicating that moderate zinc intake has a protective effect.

**Clinical trial:**

Not applicable.

## Introduction

Herpes simplex virus (HSV), a double-stranded DNA pathogen with a global prevalence, manifests as two primary serotypes: HSV-1 and HSV-2. HSV-1 spreads predominantly through oral‒to‒oral contact, infecting more than 67% of individuals under 50 years of age, whereas HSV-2 is transmitted primarily via sexual routes, affecting approximately 17% of the 15–49 age cohort [1]. Following initial mucosal invasion, the virus establishes lifelong latency in the sensory ganglia, characterized by periodic reactivation [2]. Although most outbreaks involve self-limiting mucocutaneous lesions, severe complications may arise, including viral encephalitis, herpetic keratitis (a leading cause of infectious blindness), and increased susceptibility to human immunodeficiency virus (HIV) infection [3–5]. The absence of curative vaccines or virucidal agents combined with asymptomatic viral shedding that facilitates transmission underscores the urgent need to investigate modifiable risk factors, such as dietary patterns, in HSV pathogenesis and epidemiology [6].

Zinc is a crucial trace element for human growth, development, and maintenance of immune function [7]. Its immunomodulatory properties extend to both innate and adaptive immunity, wherein it participates in lymphocyte differentiation, cytokine production, and phagocytic activity [8–10]. Zinc status serves as a critical determinant of antiviral immunity, with zinc-deficient populations demonstrating heightened susceptibility to viral infections, including HIV and hepatitis C virus [11, 12]. Moreover, emerging evidence has revealed the direct inhibitory effects of zinc on HSV replication through mechanisms involving viral DNA polymerase interference and capsid destabilization [13–15]. Although the interplay between serum zinc status and viral pathogenesis has been extensively investigated, the specific relationship between dietary zinc intake and HSV seropositivity remains unexplored.

Therefore, further research is necessary to understand the association between dietary zinc consumption and HSV seropositivity. Data from the National Health and Nutrition Examination Survey (NHANES) were drawn upon in the present study.

## Materials and methods

### Study population

The NHANES, organized by the United States Centers for Disease Control and Prevention (CDC), serves as a critical surveillance system monitoring population health indicators among noninstitutionalized U.S. residents. In accordance with the approval of the National Center for Health Statistics Research Ethics Committee, the protocol mandates documented consent from all participants prior to enrollment. For our study that analyzed existing deidentified records, no supplementary ethical clearance or informed consent was necessary according to federal regulations. Publicly accessible datasets spanning 2007–2016 (5 biennial cycles) were retrieved from the official NHANES repository (https://www.cdc.gov/nchs/nhanes). We utilized data from this five cycles because they represent the most recent consecutive cycles (relative to the present) that contain concurrent laboratory data for both HSV-1/HSV-2 and zinc intake data. Our analytical cohort excluded individuals whose nutritional zinc records were incomplete, whose serological data for herpes simplex virus were unavailable, or whose covariate information was missing. The final sample included complete case data from eligible participants across the designated survey periods.

### Dietary zinc intake

Participants in the NHANES dietary survey reported their food and beverage consumption over a 24-hour period, with data collected between 2006 and 2016 via the automated multiple pass method (AMPM) to ensure precise nutrient calculations on the basis of reported intake. The detailed methodologies are described in the NHANES Dietary Interviewer Procedure Manual. Dietary zinc intake was calculated from the first 24-h recall only (DR1TZINC). Zinc intake was analyzed as a continuous variable and categorized into quartiles (Q1–Q4). To validate these results, individuals whose reported 24-hour dietary intake aligned with their habitual consumption patterns were prioritized. Specifically, participants were screened via the variable DR1_300, which corresponds to the following question: “Was the amount of food that {you/NAME} ate yesterday much more than usual, usual, or much less than usual?” Only those who responded “usual” were included. Additionally, the zinc intake categories for Q1 were as follows: <7.51 mg/day, Q2: 7.51–10.87 mg/day, Q3: 10.87–15.63 mg/day, and Q4: >15.63 mg/day.

### HSV antibody measurement

To identify HSV-1 and HSV-2 infections, blood samples obtained through venipentesis were analyzed via a solid-phase enzyme immunopoint assay. The publicly released NHANES data file includes HSV-1 data for the 14–49 age group and HSV-2 data for the 18–49 age group. In our study, these two variables were considered independent factors; that is, HSV-1 and HSV-2 infections were defined as positive for their respective viral antibodies, and negative infections were defined as negative for their respective viral antibodies. Records classified as “inconclusive” were excluded from our analysis.

### Covariates

We evaluated several potential covariates on the basis of previous literature [16–18], including age; sex; race and ethnicity; body mass index (BMI); marital status; smoking status; excessive alcohol consumption; number of lifetime sexual partners; acquisition time; and caloric, protein, carbohydrate, sugar, fiber, and fat intake. The categories for race and ethnicity included Mexican American, other Hispanic, non-Hispanic White, non-Hispanic Black, and other races. Marital status was categorized as living alone or being married/living with a partner. BMI was calculated via a standardized approach considering both weight and height. Smoking was defined as having smoked at least 100 cigarettes. Excessive alcohol consumption was defined as periods of drinking 4 or more drinks per day for women and 5 or more for men. Sexual partners were defined as the total number of same-sex or opposite-sex partners with whom any sexual activity occurred. A dietary recall interview was conducted prior to the Mobile Examination Center (MEC) interview to collect participants’ nutritional data over a 24-hour period, including the consumption of calories, protein, carbohydrates, sugar, fiber, and fat.

### Statistical analysis

This was a secondary analysis based on publicly available NHANES datasets. Given the complex, multistage probability sampling design of NHANES, all analyses incorporated sampling weights, strata, and primary sampling units (PSUs) to produce nationally representative estimates and valid standard errors, following the analytical guidelines provided by the National Center for Health Statistics (NCHS). Data from five NHANES cycles (2007–2016) were pooled following the NCHS analytic guidelines [19, 20]. Sample weight (WTDRD1), Strata (SDMVSTRA) and PSU (SDMVPSU) were included in every SURVEY procedure (svyset in R) to obtain design-consistent point estimates and Taylor-linearization standard errors.

For continuous variables with a normal distribution, we reported the mean and standard deviation (SD). For nonnormally distributed variables, we used medians and interquartile ranges (IQRs). Categorical variables are presented as percentages. To compare differences between groups, we used one-way analysis of variance (ANOVA) for normally distributed data, the Mann-Whitney test for nonnormally distributed data, and the chi‒square test for categorical data. Logistic regression analysis was used to explore the association between dietary zinc intake and HSV seropositivity. Initially, we used univariate logistic regression to identify factors associated with HSV-1 and HSV-2 seropositivity. After controlling for potential confounding factors, we used a multivariate logistic regression model to clarify the relationships between dietary zinc intake and HSV-1 and HSV-2 seropositivity. Model I was adjusted for age, sex, and race. In Model II, we further adjusted for variables such as marital status, smoking status, excessive alcohol consumption, number of lifetime sexual partners, and acquisition time. In Model III, we included all the covariates.

Furthermore, restricted cubic spline (RCS) regression analysis was used to assess whether there was a nonlinear relationship between dietary zinc intake and HSV-1 and HSV-2 seropositivity. RCS models were fitted with the survey-weighted logistic procedure (survey::svyglm in R) using the 10-year weights described above. Four knots were placed at the 5th, 35th, 65th and 95th weighted percentiles of zinc intake. Finally, a subgroup analysis was conducted to compare the interaction between dietary zinc intake and HSV-1 and HSV-2 seropositivity across different subgroups was used to evaluate the robustness of the study results.

All statistical analyses accounted for the NHANES complex survey design and were conducted using R software (version 4.4.2; R Foundation for Statistical Computing, Vienna, Austria; http://www.R-project.org).

## Results

### Study population

**Fig. 1:**
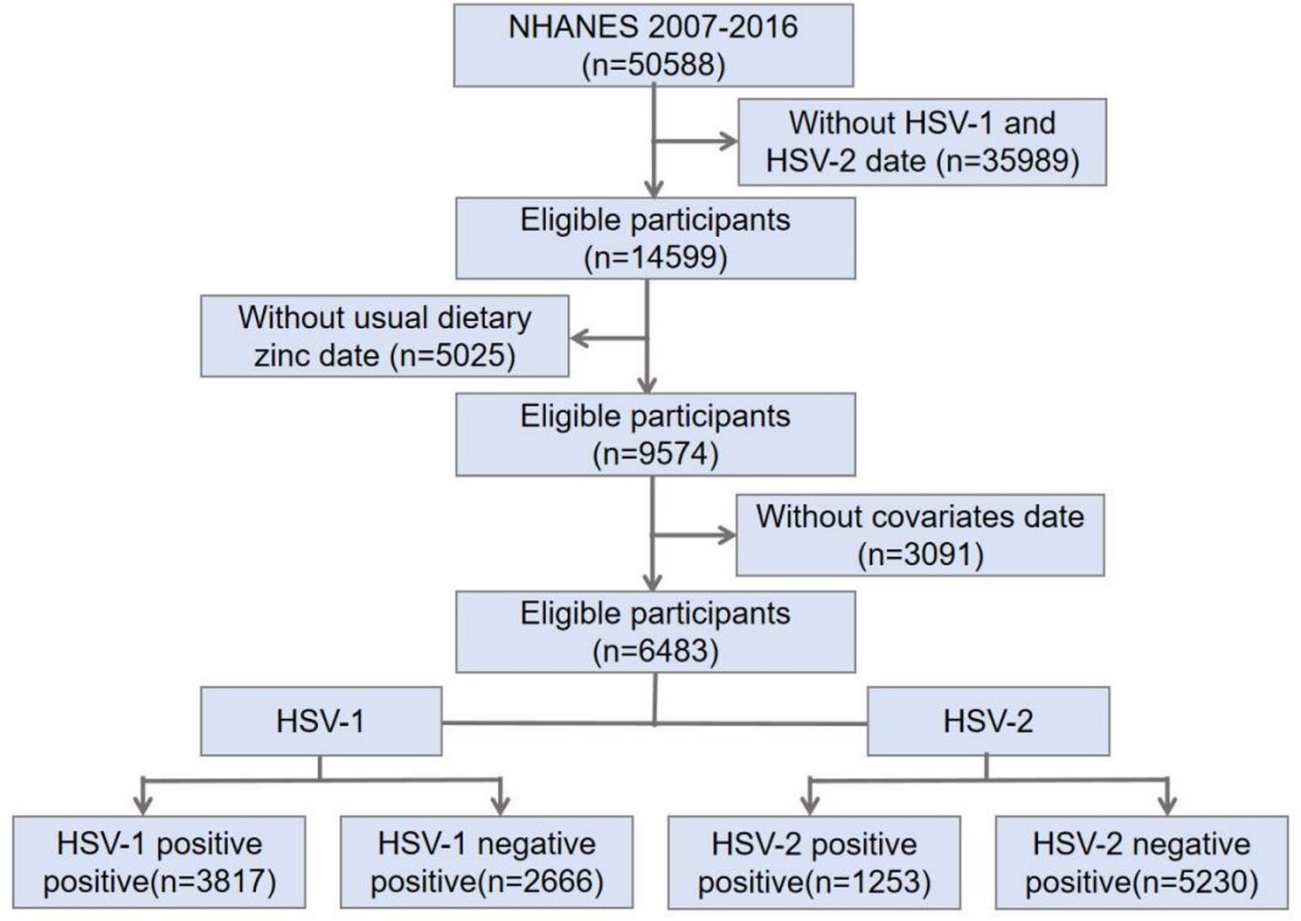
The study’s flow diagram. HSV, herpes simplex virus; NHANES, National Health and Nutrition Examination Survey

A total of 50,588 participants were included in the NHANES conducted between 2007 and 2016. After individuals with missing HSV-1 and HSV-2 data were excluded, 14,599 individuals were included in the study. After selecting those for whom data regarding dietary zinc were available, 9,574 people remained. After further screening for individuals who could recollect the food they consumed the previous day and excluding individuals with missing covariates, 6,483 people remained. The final population included 2,666 HSV-1-negative and 3,817 HSV-1-positive individuals. For HSV-2, there were 5,230 HSV-2-negative individuals and 1,253 HSV-2-positive individuals. The mean age was 34.52 ± 8.64 years, and 61.4% were male. The mean daily zinc consumption amount was 12.57 ± 8.23 mg (10.12 ± 5.42 mg for women and 14.87 ± 9.64 mg for men) (Fig. 1).

### Baseline characteristics

Table 1 summarizes the baseline demographic characteristics stratified by herpes simplex virus (HSV) infection status. Seropositivity for HSV (types 1 and 2) was associated with advanced age, elevated BMI, lower caloric intake, diminished protein and fat consumption, higher binge drinking frequency, increased lifetime sexual partners, female predominance, increased smoking prevalence, reduced zinc intake, and the highest disease incidence during the 2007–2008 survey cycle. Notably, HSV-1 seropositivity was linked to higher cohabitation rates, with the highest seroprevalence observed in Mexican American individuals. In contrast, HSV-2 seropositivity was correlated with a lower prevalence of cohabitation, the highest incidence among non-Hispanic Black populations, reduced carbohydrate and sugar intake, and an elevated frequency of sexual partners annually.

**Table 1:** Baseline characteristics and demographic characteristics of the participants

Variables	HSV-1 +(*n* = 3817)	HSV-1 -(*n* = 2666)	*P*	HSV-2 +(*n* = 1253)	HSV-2 -(*n* = 5230)	*p*
Age	37.0 (29.0, 43.0)	31.0 (25.0, 39.0)	< 0.001	39.0 (32.0, 45.0)	33.0 (26.0, 41.0)	< 0.001
Gender			< 0.001			< 0.001
Male	1874 (49.1%)	1461 (54.8%)		457 (36.5%)	2878 (55.0%)	
Femlale	1943 (50.9%)	1205 (45.2%)		796 (63.5%)	2352 (45.0%)	
Race and ethnicity			< 0.001			< 0.001
Mexican American	791 (20.7%)	214 (8.0%)		140 (11.2%)	865 (16.5%)	
Other Hispanic	436 (11.4%)	157 (5.9%)		136 (10.9%)	457 (8.7%)	
Non-Hispanic White	1528 (40.0%)	1561 (58.6%)		438 (35.0%)	2651 (50.7%)	
Non-Hispanic Black	659 (17.3%)	414 (15.5%)		464 (37.0%)	609 (11.6%)	
Other/Multi-Racial	403 (10.6%)	320 (12.0%)		75 (6.0%)	648 (12.4%)	
Marital status			< 0.001			< 0.001
Married or living with a partner	2432 (63.7%)	1521 (57.1%)		685 (54.7%)	3268 (62.5%)	
Living alone	1385 (36.3%)	1145 (42.9%)		568 (45.3%)	1962 (37.5%)	
BMI			< 0.001			< 0.001
Low(<18.5)	2669 (69.9%)	1659 (62.2%)		940 (75.0%)	3388 (64.8%)	
Median(18.5–25)	57 (1.5%)	44 (1.7%)		17 (1.4%)	84 (1.6%)	
High(> 25)	1091 (28.6%)	963 (36.1%)		296 (23.6%)	1758 (33.6%)	
Smoking status			< 0.001			< 0.001
Yes	1727 (45.2%)	1022 (38.3%)		651 (52.0%)	2098 (40.1%)	
No	2090 (54.8%)	1644 (61.7%)		602 (48.0%)	3132 (59.9%)	
Number of lifetime sexual partners			< 0.001			< 0.001
0–4	1305 (34.2%)	1045 (39.2%)		233 (18.6%)	2117 (40.5%)	
4–10	1272 (33.3%)	815 (30.6%)		467 (37.3%)	1620 (31.0%)	
>10	1240 (32.5%)	806 (30.2%)		553 (44.1%)	1493 (28.5%)	
Excessive alcohol consumption			< 0.001			< 0.001
Yes	633 (16.6%)	333 (12.5%)		231 (18.4%)	735 (14.1%)	
No	3184 (83.4%)	2333 (87.5%)		1022 (81.6%)	4495 (85.9%)	
Carbohydrate consumption(gm/day)	255.8 (188.2, 344.4)	266.2 (197.7, 358.9)	< 0.001	252.7 (180.5, 351.0)	262.1 (194.9, 350.4)	0.016
Fiber consumption(gm/day)	14.9 (9.6, 22.2)	15.3 (10.4, 22.4)	0.009	13.2 (8.7, 19.9)	15.5 (10.2, 22.9)	< 0.001
Calorie consumption(kcal/day)	2122.0 (1600.0, 2849.0)	2268.5 (1699.0, 2955.8)	< 0.001	2094.0 (1527.0, 2808.0)	2200.5 (1673.0, 2915.8)	< 0.001
Protein consumption(gm/day)	80.9 (58.4, 111.7)	84.9 (62.5, 113.9)	< 0.001	76.3 (55.0, 104.9)	84.9 (61.4, 114.5)	< 0.001
Sugar consumption(gm/day)	109.3 (67.6, 162.0)	109.1 (69.8, 163.6)	0.409	112.1 (70.0, 172.5)	108.3 (68.4, 159.6)	0.027
Fat consumption(gm/day)	77.4 (53.0, 109.5)	83.6 (58.6, 115.9)	< 0.001	78.0 (52.3, 110.3)	80.4 (56.6, 112.2)	0.008
Zinc intake (mg/day)	10.6 (7.2, 15.4)	11.2 (7.8, 16.0)	< 0.001	9.7 (6.7, 14.6)	11.1 (7.7, 15.9)	< 0.001
Zinc intake category			< 0.001			< 0.001
Q^a^1(≤ 7.505)	1026 (26.9%)	595 (22.3%)		403 (32.2%)	1218 (23.3%)	
Q^a^2(7.505–10.87)	953 (25.0%)	675 (25.3%)		307 (24.5%)	1321 (25.3%)	
Q^a^3(10.87–15.63)	919 (24.1%)	697 (26.1%)		276 (22.0%)	1340 (25.6%)	
Q^a^4(≥15.63)	919 (24.1%)	699 (26.2%)		267 (21.3%)	1351 (25.8%)	
Source			= 0.003			< 0.001
2007–2008	775 (20.3%)	450 (16.9%)		275 (21.9%)	950 (18.2%)	
2009–2010	908 (23.8%)	607 (22.8%)		315 (25.1%)	1200 (22.9%)	
2011–2012	717 (18.8%)	525 (19.7%)		251 (20.0%)	991 (18.9%)	
2013–2014	709 (18.6%)	545 (20.4%)		216 (17.2%)	1038 (19.8%)	
2015–2016	708 (18.5%)	539 (20.2%)		196 (15.6%)	1051 (20.1%)	

Baseline characteristics categorized by dietary zinc intake. ^a^Quartiles based on dietary zinc consumption

### Relationship between dietary zinc intake and HSV infection

Univariate analysis revealed that both HSV-1 and HSV-2 were associated with age; sex; race; marital status; smoking status; excessive alcohol consumption; lifetime number of sexual partners; year of data collection; and calorie intake, protein intake, dietary fiber intake, zinc intake, and zinc intake quartile groups. Additionally, HSV-1 was specifically associated with carbohydrate intake, whereas HSV-2 was associated with sugar intake (Table 2).

**Table 2:** Associations between covariates and asthma risk

Variable	HSV-1-OR^a^(95%CI^b^)	*p* Value	HSV-2-OR^a^(95%CI^b^)	*p* Value
Zinc intake category				
Q1(≤ 7.505)	Reference		Reference	
Q2(7.505–10.87)	0.82 (0.71–0.94)	0.005	0.70 (0.60–0.83)	< 0.001
Q3(10.87–15.63)	0.76 (0.66–0.88)	< 0.001	0.62 (0.53–0.74)	< 0.001
Q4(≥15.63)	0.76 (0.66–0.88)	< 0.001	0.60 (0.50–0.71)	< 0.001
Age	1.05 (1.05–1.05)	< 0.001	1.06 (1.05–1.08)	< 0.001
Gender				
Male	Reference		Reference	
Femlale	1.25 (1.14–1.39)	< 0.001	2.13 (1.89–2.44)	< 0.001
Race and ethnicity				
Mexican American	Reference		Reference	
Other Hispanic	0.75 (0.60–0.95)	0.018	1.85 (1.41–2.38)	< 0.001
Non-Hispanic White	0.26 (0.22–0.31)	< 0.001	1.02 (0.83–1.25)	0.844
Non-Hispanic Black	0.43 (0.35–0.52)	< 0.001	4.76 (3.85–5.90)	< 0.001
Other/Multi-Racial	0.34 (0.27–0.42)	< 0.001	0.71 (0.53–0.96)	0.028
Marital status				
Married or living with a partner	Reference		Reference	
Living alone	0.76 (0.68–0.84)	< 0.001	0.72 (0.64–0.82)	< 0.001
BMI				
Low(<18.5)	Reference		Reference	
Median(18.5–25)	0.88 (0.58–1.30)	0.514	0.83 (0.50–1.47)	0.501
High(> 25)	1.24 (0.83–1.86)	0.286	1.37 (0.83–2.26)	0.24
Excessive alcohol consumption, n				
Yes	Reference		Reference	
No	0.72 (0.62–0.83)	< 0.001	0.72 (0.61–0.85)	< 0.001
Smoking status				
No	Reference		Reference	
Yes	0.75 (0.68–0.83)	< 0.001	0.62 (0.55–0.70)	< 0.001
Source				
2007–2008	Reference		Reference	
2009–2010	0.87 (0.74–1.01)	0.075	0.91 (0.76–1.09)	0.294
2011–2012	0.79 (0.69–0.93)	0.005	0.88 (0.72–1.06)	0.175
2013–2014	0.76 (0.64–0.89)	< 0.001	0.72 (0.59–0.88)	0.001
2015–2016	0.76 (0.65–0.89)	0.001	0.65 (0.53–0.79)	< 0.001
Number of lifetime sexual partners				
0–4	Reference		Reference	
4–10	1.25 (1.11–1.41)	< 0.001	2.63 (2.22–3.12)	< 0.001
>10	1.23 (1.09–1.39)	< 0.001	3.33 (2.86–3.92)	< 0.001
Calorie consumption(kcal/day)	1.00 (1.00–1.00)	< 0.001	1.00 (1.00–1.00)	0.019
Protein consumption(gm/day)	1.00 (1.00–1.00)	0.009	1.00 (1.00-1.01)	< 0.001
Carbohydrate consumption(gm/day)	1.00 (1.00–1.00)	0.007	1.00 (1.00–1.00)	0.184
Sugar consumption(gm/day)	1.00 (1.00–1.00)	0.195	1.00 (1.00–1.00)	0.003
Fiber consumption(gm/day)	0.99 (0.99–0.99)	0.018	0.98 (0.97–0.98)	< 0.001
Fat consumption(gm/day)	1.00 (1.00–1.00)	< 0.001	1.00 (1.00–1.00)	0.109
Zinc intake (mg/day)	0.99 (0.99–0.99)	0.023	0.98 (0.97–0.99)	< 0.001

^a^Odds ratio. ^b^Confidence interval

In the multivariable-adjusted model evaluating the associations between zinc intake quartiles (low, low-moderate, moderate-high, and high intake) and HSV-1/HSV-2 infections, the following results were observed. Compared with the low-zinc intake group, the low-moderate intake group presented a reduced risk of HSV-1 infection, with an adjusted odds ratio (OR) of 0.84 (95% confidence interval [CI]: 0.70–0.99, *p* = 0.028), and a statistically significant reduction in HSV-2 infection risk (OR = 0.81, 95% CI: 0.66–0.99, *p* = 0.039). Moderate–high zinc intake was associated with a decreased risk of HSV-1 infection (OR = 0.84, 95% CI: 0.70–0.99, *p* = 0.042), but no statistically significant reduction in HSV-2 infection risk was observed (OR = 0.84, 95% CI: 0.68–1.05, *p* = 0.136). High zinc intake did not significantly reduce the risk of HSV-1 or HSV-2 infection (*p* > 0.05) (Table 3).

**Table 3:** Associations between dietary zinc intake and HSV

Quartile	HSV-1 OR^a^(95%CI^b^)									
	No.	*n*(%)	Crude	*p* Value	Model 1	*p* Value	Model 2	*p* Value	Model 3	*p* Value
Zincintake(mg/day)										
Q^c^1(≤ 7.505)	1621	1026(63.3%)	1(Ref)		1(Ref)		1(Ref)		1(Ref)	
Q^c^2(7.505–10.87)	1628	953(58.5%)	0.82(0.71–0.94)	0.005	0.8(0.69–0.93)	0.003	0.81(0.69–0.94)	0.006	0.84(0.72–0.98)	0.028
Q^c^3(10.87–15.63)	1616	919(56.9%)	0.76(0.66–0.88)	<0.001	0.78(0.67–0.91)	0.002	0.79(0.67–0.92)	0.002	0.84(0.70–0.99)	0.042
Q^c^4(≥15.63)	1618	919(56.8%)	0.76(0.66–0.88)	<0.001	0.83(0.71–0.97)	0.018	0.81(0.69–0.95)	0.009	0.89(0.72–1.11)	0.292
	HSV-2 OR^a^(95%CI^b^)									
Zincintake(mg/day)										
Q^c^1(≤ 7.14)	1621	403(24.9%)	1(Ref)		1(Ref)		1(Ref)		1(Ref)	
Q^c^2(7.14–10.53)	1628	307(18.9%)	0.70(0.59–0.83)	<0.001	0.83(0.69–0.99)	0.04	0.80(0.66–0.97)	0.021	0.81(0.66–0.99)	0.039
Q^c^3(10.53–15.23)	1616	276(17.1%)	0.62(0.51–0.74)	<0.001	0.86(0.71–1.04)	0.13	0.84(0.69–1.02)	0.077	0.84(0.68–1.05)	0.136
Q^c^4(≥15.23)	1618	267(16.5%)	0.60(0.50–0.71)	<0.001	0.96(0.79–1.71)	0.69	0.88(0.72–1.08)	0.237	0.90(0.67–1.19)	0.454

Model 1 was adjusted for age, sex, race and ethnicity; Model 2 was adjusted for Model 1 + smoking status, excessive alcohol consumption, sex partner, BMI, marital status, and source; Model 3 was adjusted for Model 2 + caloric consumption, protein consumption, carbohydrate consumption, sugar consumption, fiber consumption, and fat consumption. ^a^Odds ratio. ^b^Confidence interval. ^c^Quartiles based on dietary zinc consumption

**Fig. 2:**
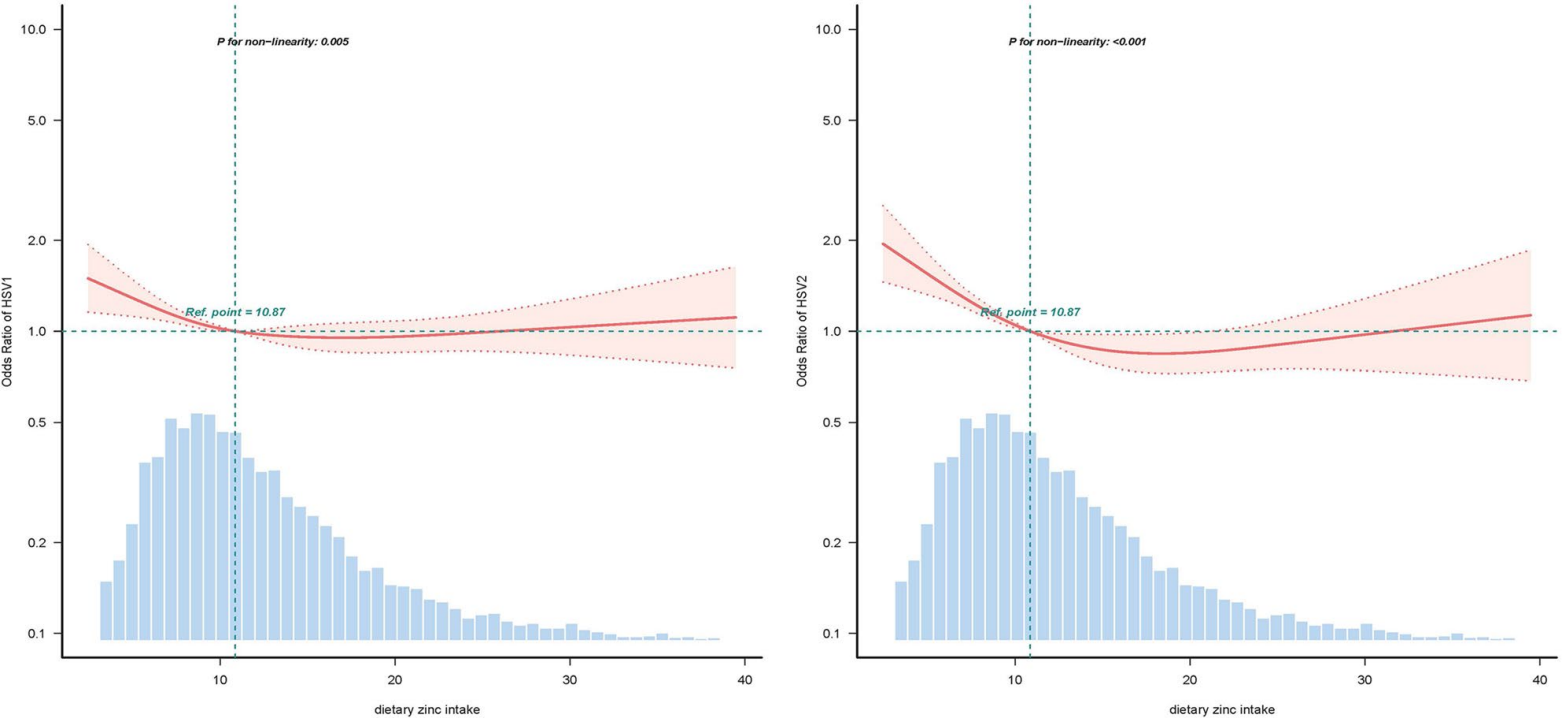
Association between dietary zinc intake and the dietary zinc intake odds ratio (HSV-1 on the left; HSV‐2 on the right). The solid and dashed lines represent the predicted values and 95% confidence intervals, respectively. They were adjusted for age, sex, race and ethnicity, smoking status, excessive alcohol consumption, sex partner, BMI, marital status, source, calorie consumption, protein consumption, carbohydrate consumption, sugar consumption, fiber consumption, and fat consumption. Only 99% of the data are shown

These results suggest that low-to-moderate zinc intake may confer a protective effect against both HSV-1 and HSV-2 infections; however, this effect diminishes or becomes insignificant at higher intake levels. Furthermore, restricted cubic spline (RCS) analysis indicated consistent U-shaped trends in the relationships between zinc intake and HSV-1 (p for nonlinearity = 0.005) and HSV-2 (p for nonlinearity < 0.01), supporting a nonlinear dose‒response pattern (Fig. 2).

### Stratified analyses based on additional variables and sensitivity analysis

**Fig. 3:**
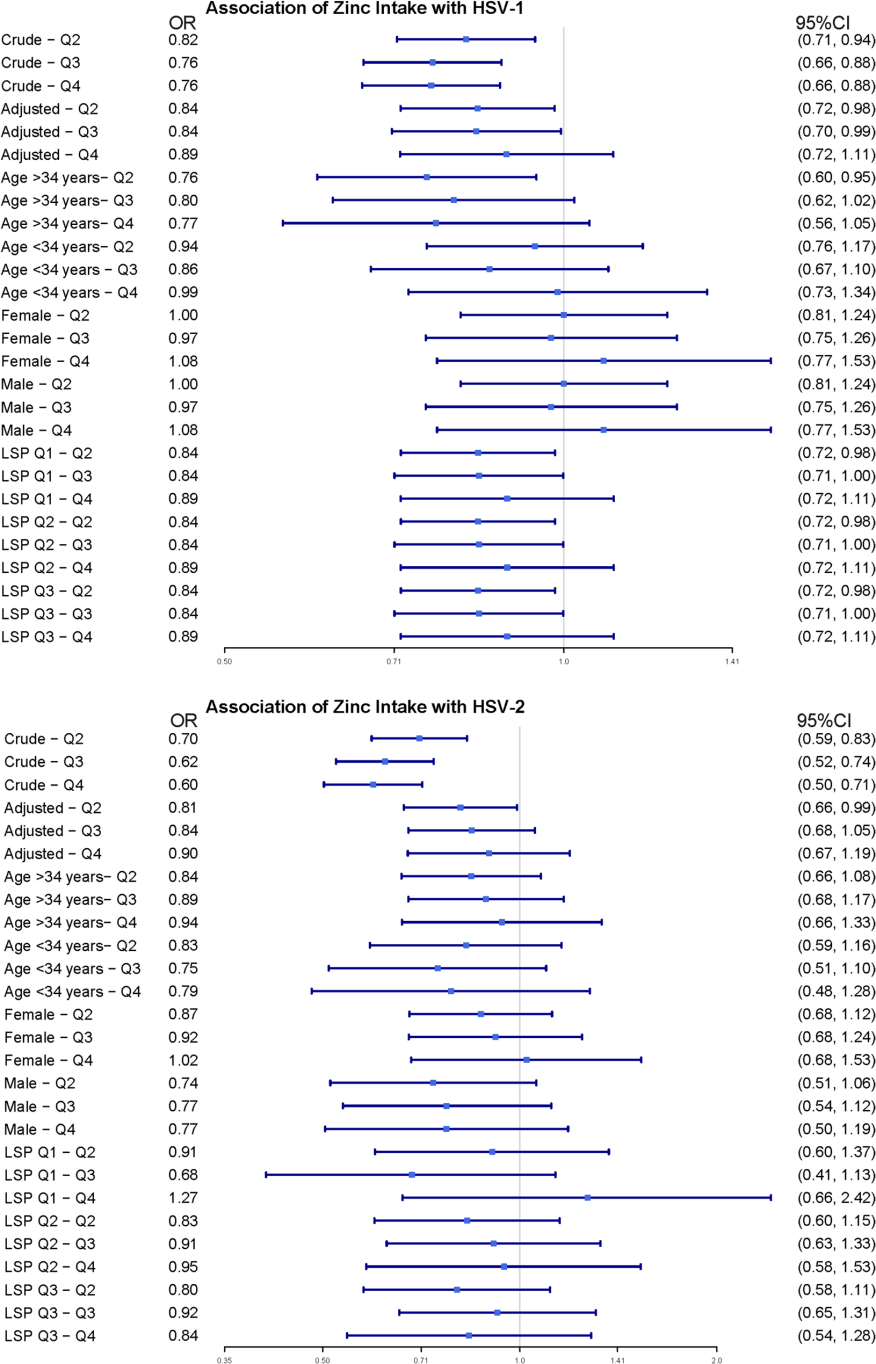
Forest plot of multivariable logistics analysis between dietary zinc intake and HSV-1 or HSV-2. Except for the stratification component itself, each stratification factor was adjusted for age, sex, race and ethnicity, smoking status, excessive alcohol consumption, sex partner, BMI, marital status, source, calorie consumption, protein consumption, carbohydrate consumption, sugar consumption, fiber consumption, fat consumption

We performed stratified analyses across multiple subgroups to assess potential variations in the association between dietary zinc intake and HSV-1/HSV-2 seropositivity. Stratification according to sex, age and the number of sexual partners revealed no additional interactions (Fig. 3). We tested the sensitivity of the model using multiple imputation, and the results showed that both HSV-1 and HSV-2 were consistent with the trends in the original model. The results are shown in Table 4.

**Table 4:** Estimated results after multiple imputation

Zincintake(mg/day)	HSV-1		HSV-2	
	estimate	*p*	estimate	*p*
Q^a^1(≤ 7.14)	reference			
Q^a^2(7.14–10.53)	-0.176	0.028	-0.208	0.039
Q^a^3(10.53–15.23)	-0.179	0.042	-0.169	0.136
Q^a^4(≥15.23)	-0.117	0.292	-0.108	0.454

^a^Quartiles based on dietary zinc consumption

## Discussion

In this cross-sectional analysis, dietary zinc intake exhibited a U-shaped statistical relationship with HSV-1/HSV-2 seroprevalence: participants in the lowest and highest intake quintiles had higher weighted seroprevalence than those in the middle quintiles. Stratified and sensitivity analyses yielded a similar pattern.

As an essential trace element in humans, zinc plays a critical role in immune regulation. Its association with HSV infection has been demonstrated in multiple studies [21–23]. In vitro experiments showed that zinc lactate inactivates more than 97% of HSV-1/2 clinical isolates and disrupts viral replication by inhibiting viral protein synthesis and potentially interfering with DNA replication [24]. Clinical trials have reported that systemic zinc supplementation (e.g., 22.5–50 mg zinc sulfate daily) significantly reduces the severity and recurrence of herpes labialis and genital herpes [25, 26].Conversely, zinc deficiency has been associated with prolonged lesion duration and impaired antiviral responses [27]. Topical zinc formulations (e.g., zinc oxide/glycine cream or 4% zinc sulfate solution) also have potential for shortening lesion duration, reducing recurrence rates (≤ 6% vs. 80% in controls), and alleviating symptoms, with no significant side effects [28, 29]. Zinc oxide nanoparticles were found to directly antagonize HSV-1 infection by suppressing viral genomic replication [30].Collectively, these findings confirm zinc’s capacity to modulate antiviral defense mechanisms against HSV.

Interestingly, the non-linear pattern observed in our study may reflect zinc’s dual immunomodulatory nature. Zinc deficiency impairs the function of innate and adaptive immune cells, such as macrophages, neutrophils, and T lymphocytes, increasing susceptibility to viral infections. However, excessive zinc intake can lead to immune dysregulation, partly through inducing copper deficiency, oxidative stress, and altered cytokine signaling [31, 32]. Similar U-shaped relationships have been proposed for other viral infections, where both zinc deficiency and excess compromise immune balance and antiviral defense [33]. However, direct evidence across different viral infections remains limited, warranting further investigation.

From a public health perspective, our results underscore the importance of balanced micronutrient intake rather than indiscriminate supplementation. Given the widespread use of zinc supplements, understanding the threshold beyond which zinc loses its protective effect is critical for refining dietary guidelines and preventing potential adverse outcomes. Future mechanistic studies should explore how zinc homeostasis interacts with specific immune pathways involved in HSV latency and reactivation.

These findings collectively confirm that zinc can directly inhibit the replication of HSV. However, excessive zinc intake also poses risks. Long-term high-dose zinc intake may lead to copper deficiency-related symptoms, including impaired immune function, reduced high-density lipoprotein levels, and elevated low-density lipoprotein levels, and neurological symptoms [34, 35]. Consequently, the protective effects of zinc against HSV require further large-scale experimental validation to establish optimal intake thresholds.

### Strengths and limitations

Our study has several strengths. First, we utilized data from multiple NHANES cycles, which substantially increased the sample size and enhanced the statistical power and generalizability of our findings. Another notable advantage lies in our focus on the continuity of dietary intake. By comparing foods consumed on a daily basis with usual intake patterns, we ensured consistency in assessing zinc consumption, making the results more reflective of real-world scenarios. In addition, we applied rigorous statistical approaches, including multivariate logistic regression, stratified analysis, and nonlinear modeling, to comprehensively explore the relationship between dietary zinc intake and HSV infection. The use of survey weights and consideration of NHANES’complex sampling design further strengthened the representativeness of our results at the population level.

However, several limitations should be acknowledged. First, this study was based on a cross-sectional design, which precludes causal inference. The observed association may also reflect reverse causation, where preexisting HSV infection could influence dietary behaviors or zinc metabolism. Second, although survey weights were applied to account for the complex NHANES design, this approach may reduce statistical precision, particularly in nonlinear spline modeling. Small sample sizes at extreme zinc intake levels may also lead to instability of the curve estimates. Third, despite extensive covariate adjustment, residual confounding cannot be entirely excluded. Furthermore, the reliance on self-reported 24-hour dietary recall data is subject to recall bias and day-to-day variability, which may affect the accuracy of zinc intake assessment. Lastly, the timing of initial HSV infection could not be determined, which may introduce temporal bias.

Future research should prioritize large-scale prospective cohort studies and multicenter randomized controlled trials to establish causal relationships and define optimal zinc intake thresholds for viral infection prevention.

## Conclusion

Our analysis revealed a U-shaped association between zinc intake and HSV-1/HSV-2 seropositivity. Although the dual antiviral and immunomodulatory properties of zinc are mechanistically plausible, causality requires confirmation in longitudinal cohorts and randomized controlled trials. If validated, zinc supplementation may emerge as a cost-effective adjunct strategy for HSV prevention.

## Data Availability

All data were sourced from the publicly available NHANES. All statistical code are available by contrating the corresponding authors with proper reason.

## Abbreviations

HSV: Herpes simplex virus
ELISA: Enzyme-Linked Immunosorbent Assay
BMI: Body Mass Index
NHANES: National Health and Nutrition Examination Survey
RCS: Restricted Cubic Splines
CI: Confidence Interval
NCHS: National Centre for Health Statistics
SE: Standard Error
